# Adherence to NCCN Genetic Testing Guidelines in Pancreatic Cancer and Impact on Treatment

**DOI:** 10.1093/oncolo/oyad044

**Published:** 2023-03-18

**Authors:** Fionnuala Crowley, Sonal Gandhi, Michelle Rudshteyn, Mantej Sehmbhi, Deirdre J Cohen

**Affiliations:** Department of Internal Medicine, Icahn School of Medicine, Mount Sinai Morningside West, New York, NY, USA; Department of Internal Medicine, Icahn School of Medicine, Mount Sinai Morningside West, New York, NY, USA; Department of Internal Medicine, Icahn School of Medicine, Mount Sinai Hospital, New York, NY, USA; Department of Internal Medicine, Icahn School of Medicine, Mount Sinai Morningside West, New York, NY, USA; Department of Gastrointestinal Oncology, Mount Sinai Hospital, New York, NY, USA; Department of Hematology Oncology, The Tisch Cancer Institute, Icahn School of Medicine at Mount Sinai, New York, NY, USA

## Abstract

**Introduction:**

National Comprehensive Cancer Network (NCCN) 2019 Guidelines recommend universal germline (GL) testing for patients (pts) with pancreatic cancer (PC), given germline mutations (gMut) can occur at a similar rate irrespective of an individual’s family history of cancer. Molecular analysis of tumors in those with metastatic disease is also recommended. We aimed to determine rates of genetic testing at our institution, factors associated with testing, and outcomes of those tested.

**Methods:**

Frequency of GL and somatic testing was examined in pts diagnosed with non-endocrine PC, with >2 visits between June 2019 and June 2021 at the Mount Sinai Health System. The clinicopathological variables and treatment outcomes were also recorded.

**Results:**

A total of 149 pts met the inclusion criteria. Sixty-six pts (44%) underwent GL testing: 42 (28%) at time of diagnosis with the remainder later in treatment. The rate of GL testing increased every year: 33% (2019), 44% (2020), and 61% (2021). A family history of cancer was the only variable associated with the decision to perform GL testing. Eight pts (12% of pts tested) had pathological gMut: *BRCA1* (1), *BRCA2* (1), *ATM* (2), *PALB2* (2)*, NTHL1* (1), both *CHEK2* and *APC* (1). Neither g*BRCA* pt received a PARP inhibitor, all except one received first-line platinum. Ninety-eight pts (65.7%) had molecular tumor testing (66.7% of patients with metastases). Two pts with *BRCA2* somatic mut did not have GL testing. Three pts received targeted therapies.

**Conclusion:**

Genetic testing based on provider discretion results in low rates of GL testing. Early results of genetic testing can have an impact on treatment decisions and trajectory of disease. Initiatives to increase testing are needed but must be feasible in real-world clinic settings.

## Teaser

Despite 2019 NCCN guidelines in many institutions, referral for germline and somatic testing is often made at the provider’s discretion. The results of this study serve as a reminder of the importance of genetic testing in pancreas cancer.

**Implications for Practice**

Cases of pancreatic cancer continue to rise, with treatments lagging behind. This article highlights why genetic testing is so important in patients with pancreatic cancer. It was found that genetic testing done at provider discretion results in low rates of testing. These results highlight the missed treatment opportunities and missed opportunities for cascade testing of family members that occur when testing is not done. This study tries to determine why providers decide to test some patients and not others. The present study includes the genetic testing experience of a diverse patient population that has not been included in other studies on this topic. The authors hope this study will serve as a reminder of the importance of genetic testing in pancreas cancer.

## Introduction

The global burden of pancreatic cancer continues to increase annually, with an estimated 62,210 cases diagnosed in the United States in 2022 and 49,830 expected deaths from the disease.^1^ It is predicted that pancreas cancer will escalate from the fourth to the second leading cause of cancer-related death in the US by 2030.^2^ The rise in incidence has been attributed to an ever aging population and an increase in predisposing conditions, including obesity and diabetes.^3^ Pancreas cancer is generally a disease of older adults, with both deaths and incident cases peaking between 65-69 years in males and 75-79 years in females.^3-5^ While there has been astounding progress in personalized targeted treatment for other malignancies with historically poor prognoses, similar breakthroughs in pancreas cancer have yet to be realized. The combination of oxaliplatin, irinotecan, fluorouracil, and leucovorin (FOLFIRINOX) and the combination of nanoparticle albumin bound paclitaxel (*Nab*-P) and gemcitabine are the standards of care first-line therapies for metastatic disease. The overall survival for patients included in the landmark studies for both these regimens was 8.5–11.1 months.^6,7^ There are a small number of targeted therapies approved by the Food and Drug administration (FDA) in pancreas cancer: EGFR inhibitor erlotinib (combined with gemcitabine) in unselected patients, *TRK* inhibitors larotrectinib and entrectinib for patients with NTRK fusion mutation, the PD-L1 inhibitor pembrolizumab for mismatch repair-deficient tumors, and the poly-ADP-ribose polymerase (PARP) inhibitor olaparib as a maintenance therapy in patients with germline *BRCA* mutation.

The accessibility of genetic testing has improved in recent years. As a result, extensive somatic and germline testing in pancreatic cancer has provided an abundance of information about the genetic landscape of the disease.^8-11^ Mutations in *KRAS, TP53, CDKN2A,* and *SMAD4* are the somatic mutations found most frequently in pancreas cancer^8,9^ with mutations in *BRCA1, BRCA2, ATM,* and *CHEK2* being the most common pathogenic germline variants.^10,11^ Up to 15% of PDAC arise in the setting of an underlying pathogenic germline mutation.^11-13^ Germline mutations in DNA damage repair (DDR) genes occur most often and patients harboring such changes benefit from a personalized treatment approach. Improved outcomes have been demonstrated with first-line platinum chemotherapy in those with mutations in homologous recombination repair (HRR) genes and with maintenance PARP inhibitors in the case of BRCA mutations.^14-17^ Other findings may inform clinical trial candidacy. One study of 854 patients with PDAC, reported that only 3 (9%) of 33 patients with a deleterious germline mutation had a significant family history of cancer. This suggests that family history is a poor predictor to determine which patients may harbor germline mutations.^18^ As a result, in 2019 the NCCN recommended that clinicians consider germline testing in any patient diagnosed with pancreas cancer and consider molecular analysis of tumors in those with metastatic disease.^19^ Despite this recommendation, implementing universal germline testing and somatic setting in metastatic disease remains an ongoing challenge.^20,21^

Some studies have examined methods to increase rates of germline genetic testing. Walker et al. found that implementation of a systematic patient intake workflow and in-clinic genetic testing station resulted in an increased rate of germline testing for patients with pancreatic cancer (from 19% to 71%).^22^ They also found that the rate of pathogenic variant detection increased from 20% to 33%.^22^ Chittenden et al. compared the rate of genetic counseling(GC)/multi-gene germline testing (MGT) before and after the implementation of automated referrals.^23^ Compared with baseline ­clinician-­directed referrals, implementation of automated referrals led to a significant increase in patients with pancreas cancer undergoing GC/MGT (16.5% vs. 38.0%, *P* < .001), including those undergoing MGT ≤ 7 days of initial oncology evaluation (14.7% vs. 60.3%, *P* < .001), with preserved pathogenic variant detection rates (10.0% vs. 11.2%, *P* = .84).^23^ Furthermore, 16 of 28 (57.1%) pathogenic variant carriers had relatives who pursued cascade germline testing, and 13 of 26 (50.0%) carriers with the incurable disease received targeted therapy based on MGT results.^23^

Referral for germline and somatic testing at our institution is currently made at the provider discretion. We aimed to determine rates of genetic testing, factors associated with testing, and outcomes of those tested.

## Methods

The project was approved by the local Institutional Review Board (IRB). Patients diagnosed with exocrine pancreas cancer between June 2019 and June 2021 in the New York Mount Sinai Health System were identified using the Mount Sinai Data warehouse. Patients who were not evaluated by medical oncology at our institution were excluded. Patients who had less than 2 visits were also excluded. The electronic record of all patients was then reviewed with the following information recorded: demographics, comorbidities, family history, stage, site(s) of disease, treatments, pathological reports, and genetic testing results. Both germline and somatic testing results were included. The company used for genetic testing varied depending on patients’ insurance. If no somatic tumor tissue results were available, ctDNA profiling from liquid biopsy samples was included. Responses were assessed according to Response Evaluation Criteria in Solid Tumors (RECIST) criteria 1.1. Family history of cancer for this article was any patient who had a documented family history of cancer in the electronic medical record. Family history of DNA damage repair (DDR) related cancer was any patient who had a documented family history of pancreatic, breast, ovarian, and/or prostate cancer in the electronic medical record.

We used descriptive statistics to report on this cohort of patients. Continuous variables were described by group means with SDs, and categorical variables were described by counts and proportions. For continuous variables, the statistical significance of differences between sample means was determined using a two-sided Student’s *t*-test, with alpha set at 0.05. For categorical variables, the association was tested with chi-squared tests when the sample size for that variable was large, and with Fisher’s exact tests when the sample size was small (<15). Statistical analysis was performed using R (version 3.6.1) in RStudio.

## Results

When all inclusion criteria were applied, 149 patients were included in the analysis. Seventy-one patients (47.7%) were male and 78 (52.3%) were female. The median age was 68 years. Detailed demographic information is included in Table 1. Reflecting the diverse population at Mount Sinai Health System, 40 patients (26.8%) were black, 44 (29.5%) were white, 19 (12.8%) were Asian, and 34 (22.8%) were Hispanic. At diagnosis, 114 patients (77%) had an ECOG performance status of 0–1. Patients had various comorbidities at diagnosis outlined in Table 1. Twenty-five (16.8%) had a history of concurrent cancers with breast (4 patients) and prostate (3 patients) being the most common. Ninety-three (62.4%) had a family history of any cancer, 49 patients (32.9%) had a family history of DNA damage repair (ovarian, prostate, breast, and pancreatic) associated cancers. Fourteen (9.4%) were Jewish and 3 patients had documented Ashkenazi heritage.

**Table 1.** Demographic features of the patient population.

	All patients *n* = 149 (%)	No GLT *N* = 83	GLT *N* = 66	*P*-value
Median age (years)	68			
Mean age (SD)		67.76 (10.56)	65.03 (11.97)	.142
ECOG				.319
ECOG performance status ≤1	114 (77)			
ECOG 0	59 (39.6)	32(38.6)	27(40.9)	
ECOG 1	55 (36.9)	27(32.5)	28(42.4)	
ECOG 2	23 (15.4)	14(16.9)	9(13.6)	
ECOG 3	7 (4.7)	6 (7.2)	1(1.5)	
Unknown	5 (3.4)	4(4.8)	1 (1.5)	
Sex				
Male	71 (47.7)			
Female	78 (52.3)			
Race/ethnicity				.796
Non- Hispanic black	40 (26.8)	21(25.3)	19(28.8)	
Non-Hispanic white	44 (29.5)	22(26.5)	22(33.3)	
Hispanic	34 (22.8)	21(25.3)	13(19.7)	
Asian	19 (12.8)	12(14.5)	7(10.6)	
Other ethnicity	12 (8.1)	7(8.4)	5(7.6)	
Ashkenazi Jewish	3 (2.0)			
Family h/o cancer	93 (62.4)	44(53)	49(74.2)	.013
Family h/o DDR pathway cancers	49 (32.9)	22(26.5)	27(40.9)	.092
Prostate	5 (3.4)			
Pancreatic	14 (9.4)			
Breast	17 (11.4)			
Endometrial	2 (1.3)			
Ovarian	3 (2.0)			
Colorectal	13 (8.7)			
FDR or SDR with pancreatic cancer	22 (14.8)			
Personal history of non-pancreatic cancer	25 (16.8)			
Breast	4 (1.6)			
Prostate	3 (1.2)			
Ovarian	1 (0.4)			
Endometrial	2 (0.8)			
Stomach	1 (0.4)			
Lung	2 (0.8)			
Lymphoma/leukemia	1 (0.4)			
Thyroid	1 (0.4)			
Renal	1 (0.4)			
Bladder	2 (0.8)			
Prostate and lung	1 (0.4)			
Skin	2 (0.8)			
Testicular	1 (0.4)			
Gallbladder	1 (0.4)			
Vulvar	1 (0.4)			
Tonsillar	1 (0.4)			
Comorbidities:				
Diabetes mellitus	55 (37.0)			
Hypertension	93 (62.4)			
Hyperlipidemia	64 (43.0)			
CAD	29 (19.5)			
Thyroid disorder	12 (8.1)			
HIV	4 (2.7)			

Abbreviations: CAD, coronary artery disease; DDR, DNA damage repair; ECOG, Eastern Cooperative Oncology Group; FDR, first degree relative; h/o, History of; SDR, second degree relative; GLT: germline testing.

The clinical stage of all patients at diagnosis is outlined in Table 2. Sixty patients (40.3%) were diagnosed with metastatic disease. One hundred and forty six patients (98%) had adenocarcinoma histology, 1 patient had squamous histology and 2 had adeno-squamous histology. Pathological variables are summarized in Table 2. Available *HER2* and mismatch match repair status results from pathology reports are also included in Table 2. Sixty-six pts (44%) had germline testing: 42 (28%) at the time of diagnosis with the remainder later in treatment. The rate of germline testing increased every year: 33% (2019), 44% (2020), and 61% (2021). There was one documented patient refusal of germline testing. The rate of germline testing was 50% in White patients, 47.5% in Black patients, 31.7% in Hispanic patients, and 36.8% in Asian patients. There was no statistically significant association between the decision to perform germline testing and age, ECOG score, race, or personal history of cancer. A family history of cancer was positively associated with germline testing (53% vs. 74.2% *P* = .013).

**Table 2.** Clinicopathological and treatment variables in the patient population.

	All patients *n* = 149 (%)
Clinical stage at diagnosis	
Resectable	28 (18.8)
Borderline resectable	31 (20.8)
Locally advanced	30 (20.1)
Metastatic	60 (40.3)
Histology	
Adenocarcinoma	146 (98.0)
Squamous	1 (0.7)
Adeno-squamous	2 (1.3)
Differentiation	
Well differentiated	2 (1.3)
Well-moderately differentiated	4 (2.7)
Moderately to poorly differentiated	26 (17.4)
Poorly differentiated	30 (20.1)
Moderately differentiated	50 (33.6)
HER2 overexpression	
Tested	75 (50.3)
Positive	16 (10.7)
Negative	59 (39.6)
Not tested/unavailable	74 (49.7)
MSI high/ MMR deficient tumor	
Yes	0
No	96 (64.4)
Unavailable	53 (35.6)
1st line platinum chemotherapy	82 (55)
Oxaliplatin	80 (53.7)
Cisplatin	2 (1.3)
Surgical treatment	33 (22.1)
Neoadjuvant treatment	22 (14.8)
Adjuvant treatment	20 (13.4)
First-line treatment for inoperable disease	114 (76.5)
FOLFIRNOX/mFOLFIRNOX	49 (42.9)
Gemcitabine plus nab-paclitaxel	41 (36)
Gemcitabine plus cisplatin	2 (1.8)
Gemcitabine alone	7 (6.0)
FOLFOX	5 (4.4)
Capecitabine	2 (1.8)
CAPIRINOX	4 (3.5)
CAPOX	2 (1.8)
Clinical trial	2 (1.8)
Outcome of first-line treatment	
Progression, transition to 2nd line	27 (18.1)
Intolerance, transition to 2nd line	7 (4.7)
Ongoing treatment	15 (10.1)
Progression, transition to supportive care	16 (10.7)
Intolerance, transition to supportive care	49 (32.9)
Targeted treatment	3 (2.0)
Immunotherapy	1 (0.7)
Radiation therapy to primary site	31 (20.8)

Eight patients (12% of those tested) had pathological germline mutations: *BRCA1* (1 patient), *BRCA2* (1 patient), *ATM* (2 patients), *PALB2* (2 patients)*, NTHL1* (1 patient), both *CHEK2* and *APC* (1 patient). Six of these patients (75%) had germline testing at diagnosis. The median age of those with germline mutations was 62.5 years. Six (75%) of those with germline mutations had a family history of cancer. Half were metastatic at diagnosis. Further information about each patient with a germline mutation is included in Table 3. Of the 7 patients with germline mutations in DDR genes, 4 had first-degree relatives with pancreas, breast, or prostate cancer. Six patients (85.7%) received platinum therapy first-line, with one patient receiving gemcitabine-*nab*-paclitaxel. The patient with germline *BRCA2* mutation was the only one to have a platinum response (partial). This patient later progressed on FOLFIRNOX and therefore did not receive a maintenance PARP inhibitor. Six patients had germline variants of unknown significance (VUS). Twenty-one patients in total had a partial response to platinum, 15 (71.4%) of these patients had germline testing. Three patients had a complete response to platinum, of whom only one had germline testing. All patients with germline mutations were referred to genetic counseling which was conducted by phone. Two patients could not be contacted by the genetic counselors. While recommended by genetic counseling, cascade testing by family members could not be determined based on the available medical records.

**Table 3.** Characteristics of patients with germline mutations

Patient	Age at dx	Gene	Mutation	Personal Hx	Family hx	Stage
1	73	ATM	c.1076_1096del21insTGTAAGG	Prostate	Yes	Borderline resectable
2	59	BRCA2	c.5946del	Endometrial	Yes	Metastatic
3	66	ATM	p.R1618*	No	Yes	Metastatic
4	40	BRCA1	EX11del	No	No	Resectable
5	45	PALB2	p.Y551	No	Yes	Borderline resectable
6	66	PALB2	c.2730T>A, p.Y910X	No	No	Locally advanced
7	58	CHEK2, APC	p.I1307K, p.S428F	No	Yes	Metastatic
8	78	NTHL1	c.268C>T(pG1n90*)	No	Yes	Borderline resectable

Ninety-two patients (61.7%) had molecular tumor testing and 6 patients had serum circulating tumor DNA (ctDNA) testing. A further 11 patients who did not have molecular results had molecular testing sent but had inadequate tissue samples for testing. Forty (66.7%) patients with metastatic disease had tumor molecular testing. Tumor samples tested most often were primary pancreatic lesions (67 patients), followed by liver samples (10 patients), and lung (2 samples). Eight patients had additional molecular testing done during the treatment course. Pathogenic mutations in *KRAS* (83 patients), *TP53* (66 patients), and *CDKN2A* (18 patients) were identified most commonly. Pathogenic mutations in DDR genes were identified in 9 patients: *ARID1A* (*n* = 2), *BRCA2* (*n* = 3), *ATM* (*n* = 2), *PALB2* (*n* = 2), and *CHEK2* (*n* = 1). Four of these had corresponding germline mutations. One patient with somatic *ATM* mutation had a germline *BRCA1* mutation. Two patients with *BRCA2* somatic mutations did not have germline testing. Variant allele frequency (VAF) of BRCA mutation in these samples was 55% and 75%, respectively. One patient had *ATM* and *CHEK2* mutations identified in ctDNA drawn 7 months after tumor molecular testing without these alterations. Forty-two patients (45% of those tested) had VUS. Aside from the mutations in the above DDR genes, only 2 other patients had targetable somatic mutations: one with a BRAF mutation and one with an IDH2 mutation.

Of the 82 patients who received platinum first-line therapy, 80 (97.6%) received oxaliplatin and 2 (2.4%) received cisplatin. Thirty-three (22.1%) had surgical resection of the primary tumor. Eleven (33.3%) of those received neoadjuvant treatment only, 9 (27.3%) received adjuvant treatment only and 11 (33.3%) received perioperative chemotherapy. FOLFIRONOX/mFOLFIRNOX(49 patients, 43%) was the regimen used most frequently in the first-line setting in the 114 patients with inoperable disease who were treated, followed by gemcitabine/*nab*-paclitaxel (41 patients, 36%). Three patients were referred to targeted clinical trials, 2 to *HER2* amplified targeted studies, and one to a study of an *IDH1* targeted agent. In the setting of inoperable disease, 65 patients (57%) did not receive second-line treatment after progression or intolerance to first-line treatment. Forty-one patients (27.5%) were still alive at data analysis, 60 (40.3%) were dead, and the current status of the remaining patients is unknown.

## Discussion

Additional effective treatment options remain an unmet need for pancreas cancer patients. Our study reinforces the results of previous studies and highlights that despite the change in NCCN guidelines three years ago, the implementation of universal germline testing and somatic testing in metastatic disease remains a challenge.

We found that rates of germline testing increased every year without implementation of an automated referral system yet still remained sub-optimal at 61%. Two studies investigating methods to increase rates of germline testing reported improved rates of testing when systematic workflows were introduced (from 19% to 71%^22^ and from 16.5% to 38.0%^23^). In health-care systems where resources are scarce, staffing is limited and there is a worldwide shortage of geneticists it is difficult to envision how workflows that require dedicated staff and designated genetic counselors would be practical in real-world clinics.^24,25^ It has also been found that genetic counsellor models which necessitate genetic counselor input prior to testing create barriers to testing especially for those with lower incomes.^26^ “Mainstreaming” genetic testing is an approach that has been studied in a number of different cancer types.^27-29^ This involves pre-test informed consent in the oncology clinic with a member of the oncology team with post-test genetic counseling for those found to have a pathogenic or likely pathogenic germline variant in a cancer susceptibility gene.^28,30^ A recent study by Ramsey et al. evaluated the implementation of mainstreaming in the pancreatic cancer cohort.^28^ They found that this program increased genetic testing among patients 6.5-fold. Even with this program, the rate of germline testing during the study period was 66.4%.^28^ Hamilton et al. examined the implementation of a mainstreaming model in prostate, pancreas, and ovarian cancer. In this study, 9.9% of patients refused germline testing.^27^ Although we are limited by information recorded in the medical record, we only had one documented refusal in our cohort. Bokkers et al. evaluated the experience of healthcare professionals with a mainstreaming approach to germline genetic testing in ovarian cancer.^30^ This article highlights some potential barriers to offering genetic testing in the clinic including concerns about the process being time consuming and the healthcare workers’ insecurity about their knowledge of genetic testing.^30^ The most common reason why genetic testing was not discussed was that the provider forgot to discuss it. Other reasons included: DNA test already being discussed, provider feeling it was not his/her role, patient had no family history of cancer, patient being too emotional, patient being too ill, and lack of time during the consultation.^30^ These insights highlight the real-world limitations to universal testing in the clinic: a lack of education around who should be tested, lack of time during consultations, cancer patients having emotional and medical needs which take priority (especially during initial consultations) with genetic testing likely forgotten as a result. At our institution, we plan to educate our oncology clinic staff again on the importance of germline testing in pancreatic cancer as well as work with our IT colleagues to establish best practice reminders for genetic testing in the electronic medical record which must be addressed before clinic encounters can be closed.

Our study also includes a very diverse patient population that has not been well represented in previous studies. Only 29.5% of our patients are white compared to 92% of patients in a similar study by Chittenden et al.^23^ We did not find any difference in rates of germline testing between races in our cohort. Disparities in genetic testing and care have been well documented across a number of different cancer types in other institutions.^31-36^ Some of these studies highlight that non-White patients are hugely underrepresented in the datasets used to evaluate genetic testing in addition to being underrepresented in clinical trials involving germline testing.^34,37-39^ Non-White patients are also underrepresented in studies examining disparities in genetic care. Liu et al. found that Black patients were less likely to complete genetic counseling when referred but only 6% of the cohort were Black.^33^ Weise et al. outlined a number of hypotheses to explain disparities of germline testing in prostate cancer including differences in quality of care for minority patients, medical mistrust, lack of knowledge regarding testing, prohibitive cost, and lack of insurance coverage.^34^ Mitigating these factors is likely the key to improving genetic care among minority populations. Hamilton et al found that black patients refused to enroll in their germline testing study at a higher rate than white patients (27% refusal rate vs. 7%).^27^ Reasons for refusal were not ntelucidated as part of this study. It is not clear to us why there was no significant difference in testing by race at our institution but additional efforts must be undertaken to identify and unravel the barriers to germline testing for minority populations generally.

Chittenden et al. found that when germline testing rates increased, the rate of pathogenic variant detection rate was preserved (10.0% vs. 11.2%, *P* = .84).^23^ Walker et al. found that with increased testing the pathogenic variant detection rate increased (from 20% to 33%). This suggests that when testing is done at provider discretion, a significant number of germline mutations may be missed. Our germline mutation rate of 12% in those tested was similar to the incidence reported in other series.^11,13^ However, it is likely that this is an under-representation of germline mutations in our population due to our low rate of testing. Interestingly, we found that having a family history of cancer was the only variable associated with the decision to perform germline testing when left to provider discretion perhaps highlighting the incorrect belief that only those with a family history are likely to have a germline mutation. In our study, six (75%) of those with germline mutations had a family history of cancer. This is higher than other studies which have shown that a large percentage of patients with germline mutations do not have a family history of cancer.^18^ Thirty-nine patients (65%) with metastatic disease had tumor molecular testing. Inadequate tissue was a barrier to testing with 11 patients having inadequate samples for testing. Six patients had ctDNA sent as a surrogate for tumor molecular testing. CtDNA testing in pancreatic cancer has been found to be a useful, safe method of assessing mutational burden, especially in metastatic cancer where mutational signature may differ depending on the site(s) of the disease biopsied.^40-42^

Both germline and somatic testing are essential as we try to advance and personalize the treatment of pancreatic cancer. Currently, there are targeted treatment options for a handful of mutations in this disease, but identifying eligible patients relies on universal testing. It is well established that patients with both germline and somatic mutations in HRR genes benefit from first-line platinum chemotherapy with stark differences (23.8 vs 8.3 months^43^) in survival between those with HRR mutations treated with platinum and those who are not.^16,43,44^ While many patients may not be able to tolerate first-line FOLFIRONOX, gemcitabine/cisplatin has been shown to be an effective regimen in advanced germline *BRCA/PALB2*+ pancreas cancer and may be a good alternative to FOLFIRNOX.^15^ Seventy-five percent of our patients with germline mutations had germline testing at diagnosis. If testing is done later in treatment and a patient is treated with a non-platinum regimen, this may represent a missed opportunity to maximize survival with first-line platinum treatment. PARP inhibitors are approved as maintenance treatment for patients with germline *BRCA* mutations. Our one potentially eligible patient progressed on FOLFIRNOX and therefore did not receive a PARP inhibitor. Four of our patients with somatic DDR mutations had corresponding germline mutations. Two patients with somatic *BRCA* mutations did not have germline testing, both with high VAF. The decision not to do germline testing in these patients may have represented a missed therapeutic opportunity for treatment with a maintenance PARP inhibitor. It may also have been a missed opportunity for cascade testing for families. Somatic testing is important for the small percentage of patients with *NTRK* fusions, *KRAS G12C* mutations, or *KRAS* wild-type patients who may have other targetable activating pathways.

Assessing for clinical trial candidacy is also an important benefit of genetic testing. Notably, following first-line treatment, the majority (57%) of our patients with inoperable disease went on to best supportive care rather than additional treatment. This is a similar number to other studies. In the landmark PRODIGE study evaluating FOLFIRNOX vs. gemcitabine alone study only 46.8% (80 patients) received second-line treatment.^6^ This highlights the need for patient enrollment in more upfront clinical trials in this disease. Only 3 of our patients were referred to a targeted therapy trial. Eligibility of one of these was decided based on molecular testing, the remaining on HER2 immunohistochemistry. Chittenden et al. found that in their institution 13 of 26 (50.0%) patients with genetic mutations and incurable diseases received targeted therapy based on MGT results.^23^ They did not specify what targeted treatments patients received or whether they received them on or off clinical trials.

Genetic testing is expensive. No studies to date have examined the cost-effectiveness of genetic testing in pancreas cancer. However, numerous analyses have consistently demonstrated that the cost-effectiveness of systematic screening for various inherited cancer syndromes is reliant on the uptake of cascade testing among at-risk relatives.^45-47^ We know that all our patients who saw clinical genetics were offered cascade testing. However, it is unclear how many families decided to proceed with the recommendation.

This study was limited by its retrospective nature and the fact that we could only extract data that was documented in the medical chart. While all genetic testing was done by approved genetic testing companies, the platform used varied among patients depending on insurance status. In addition, official RECIST imaging response reviews by a radiologist was not completed as part of this study. As discussed above in the future, we aim to add steps to our clinic workflow to increase the rates of germline testing for all newly diagnosed patients and somatic testing for patients with metastatic disease.

## Conclusion

Our study supports the results of previous studies and underscores the challenges of universal germline testing and somatic testing in metastatic disease. Early results of genetic testing can have a significant impact on treatment decisions and patient outcomes. With a growing number of targeted drugs being investigated in clinical trials, the need for genetic testing will only become more critical. However, progression or intolerance to first-line treatment remains a barrier for existing approved targeted treatments and clinical trial candidacy. Initiatives to increase testing are needed but must be feasible in real-world clinic settings. While we did not identify differences in germline testing by race additional efforts must be undertaken to identify and unravel the barriers to germline testing for minority populations generally.

## Data Availability

The data underlying this article will be shared on reasonable request to the corresponding author.

## Previous Presentation

Previously presented as an abstract at the 2022 ASCO Annual Meeting.
